# Parathyroid Carcinoma

**DOI:** 10.1359/jbmr.081018

**Published:** 2008-10-20

**Authors:** Claudio Marcocci, Filomena Cetani, Mishaela R Rubin, Shonni J Silverberg, Aldo Pinchera, John P Bilezikian

**Affiliations:** 1 Department of Endocrinology and Metabolism, University of PisaPisa, Italy; 2 Department of Medicine and Pharmacology, Columbia University, College of Physicians & SurgeonsNew York, New York, USA

## INTRODUCTION

Parathyroid carcinoma is a rare endocrine malignancy. It accounts for <1% of cases of sporadic primary hyperparathyroidism (PHPT) and is usually associated with more severe clinical manifestations than its much more common benign counterpart, parathyroid adenoma.(1–3) Its course is typically indolent but progressive. The diagnosis of malignancy is often made only when local recurrence or metastases occur, because the histology of parathyroid tumors can be equivocal or frankly misleading.(4) Most patients with recurrent disease ultimately succumb to the effects of hypercalcemia rather than to direct tumor invasion or distant metastases. A complete resection of all malignant tissue at the time of initial surgery allows for the greatest likelihood of a cure. Clinical clues to the possibility of a parathyroid cancer, therefore, should lead the surgeon to an aggressive initial operative approach. In the last decade, greater knowledge of the molecular pathogenesis of parathyroid carcinoma has led to the development of diagnostic markers that show promise, particularly when the histology is ambiguous.(2,3) Moreover, there is hope that greater understanding of the pathogenesis of parathyroid cancer will lead to the development of new therapeutic strategies for advanced, inoperable disease. This review focuses on the more recent advances in parathyroid carcinoma, particularly its molecular pathogenesis, diagnosis, and management.

## EPIDEMIOLOGY

To date, >400 cases of parathyroid carcinoma have been reported.(5) It is usually a sporadic disease, but familial cases have been described. The largest series comes from the National Cancer database.(6) In most series of PHPT, parathyroid carcinoma accounts for <1% of all cases, but an incidence as high as 5% has been reported.(1,7,8) The use of varying criteria for its pathologic diagnosis is most likely the reason why later studies have shown higher incidence rates.

In contrast to benign parathyroid disease, where women predominate over men by a ratio of 3–4:1, parathyroid cancer occurs with equal frequency in both sexes. The age at diagnosis is 10 yr earlier than the typical age when the benign form of PHPT presents (mid-40s versus mid-50s).

## ETIOLOGY

The etiology of parathyroid carcinoma is largely unknown. A potential role for prior neck irradiation is less clear than in the development of benign parathyroid disease.(9–13) Rarely, parathyroid carcinoma has been reported in patients with longstanding secondary hyperparathyroidism.(14,15) In the few such cases, it is unclear whether the currently accepted pathological criteria of parathyroid malignancy were met. Parathyroid carcinoma has also been reported in hereditary syndromes of hyperparathyroidism,(16–19) particularly in hyperparathyroidism-jaw tumor (HPT-JT) syndrome,(20) a rare autosomal disorder, in which as many as 15% of patients will have malignant parathyroid disease. Because cystic changes are common, this disorder has also been referred to as cystic parathyroid adenomatosis.(21) In HPT-JT, ossifying fibromas of the maxilla and mandible are seen in 30% of cases. Less commonly, kidney lesions, including cysts, polycystic disease, hamartomas, or Wilm's tumors, can be present.(22) Parathyroid carcinoma has also been reported in familial isolated hyperparathyroidism.(17,23) Recently, parathyroid carcinoma, as defined pathologically, has been reported in multiple endocrine neoplasia type 1 (MEN1) syndrome and with somatic *MEN1* mutations.(24–26) However, recurrent parathyroid disease in MEN1 may mimic, but not actually be caused by, malignancy. Only one case of parathyroid carcinoma has been reported in the MEN2A syndrome.(27)

## PATHOGENESIS

Oncogenes and tumor suppressor genes have been linked to parathyroid carcinomas, especially those involved in the control of the cell cycle. Examples include *retinoblastoma (Rb), p53, breast carcinoma susceptibility (BRCA2), and cyclin Dl/parathyroid adenomatosis gene 1 (PRAD1)* genes.(28,29) To date there is no definitive evidence for a primary role of these genes in parathyroid carcinoma, although altered expression of these gene products may participate in the process of malignant transformation.

After the report of Cryns et al.,(30) showing loss of Rb protein expression in parathyroid carcinoma, the absence of this protein in parathyroid tumors was proposed as a tool for the diagnosis of parathyroid malignancy. Since then, however, conflicting results have been reported by other investigators in terms of whether *Rb* gene alterations are specific for parathyroid cancer.(31–33) Moreover, Shattuck et al.(34) failed to detect microdeletions, insertions, or point mutations in the coding and promoter regions of the *Rb* gene in parathyroid carcinomas.

**FIG. 1:**
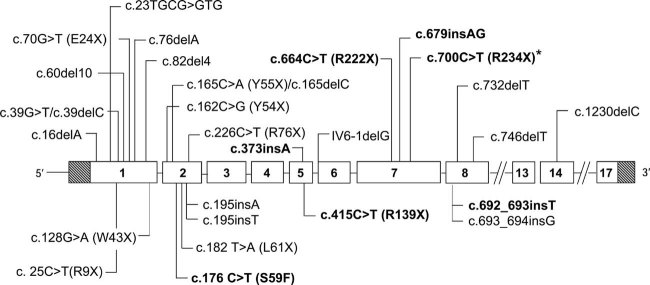
*HRPT2* mutations in sporadic parathyroid carcinomas. Mutations are designed according to the format generally used in the *HRPT2* literature with single-letter amino acid codes. Mutations in bold are germinal.(36,37,45,46) *This mutation has been found in three unrelated kindreds.(36,37)

The HPT-JT syndrome has provided the best evidence for a defined gene in parathyroid cancer. The gene is known now as *HRPT2* but other terms have been used, such as *CDC73* and *Clorf 28*.(22) Evidence points to a strong association between *HRPT2* mutations, the gene responsible for HPT-JT, and parathyroid carcinoma.(35–37) Parathyroid carcinoma occurs with higher frequency in HPT-JT than in sporadic PHPT (15% versus <1%). Similar germline mutations occur in a subset of kindreds with familial isolated hyperparathyroidism.(22,35,37–44) The role of the *HRPT2* gene in the pathogenesis of sporadic parathyroid carcinoma was shown by Howell et al.(35) and Shattuck et al.(36) in 2003. In the former study,(35) *HRPT2* mutations were detected in 4 of 4 sporadic parathyroid carcinomas and in 0 of 25 sporadic parathyroid adenomas. In the later study,(36) *HRPT2* mutations were found in 10 of 15 patients with apparently sporadic parathyroid carcinoma. Cetani et al.(37,45) identified *HRPT2* mutations in 9 of 11 parathyroid carcinomas but in 0 of 4 sporadic atypical adenomas. Most of the mutations are of the nonsense form and are predicted to result in lack of or reduced protein expression of the encoded parafibromin protein (see below) (Fig. 1). The prevalence of *HRPT2* mutations in sporadic parathyroid carcinomas may be as high as 76.6% (Table 1). Thus, the strong association between *HRPT2* mutation and parathyroid malignancy suggests that this molecular event is involved in the pathogenesis of most sporadic parathyroid carcinomas. Of particular interest is the demonstration that germline mutations were identified in about one third of subjects.(36,37,45) This finding suggests that a subset of patients with apparently sporadic parathyroid carcinomas may have the HPT-JT syndrome or a variant. Inactivating mutations of noncoding or regulatory regions could also be implicated in the pathogenesis of sporadic parathyroid carcinoma and might be present in those without alterations in the coding regions of the gene. A recent study by Haven et al.(46) found *HRPT2* inactivating mutations in only 4 of 28 (15%) cases of parathyroid carcinomas. Two mutations were germinal. These tumors were classified as malignant on the basis of pathological criteria alone without the requirement for malignant behavior. In this study, the exons that harbor 85% of all known mutations (1, 2, and 7) were completely screened. The low mutation frequency could be explained in part by the fact that not all exons could be completely evaluated because of the nature of the formalin-fixed–embedded tissue. Another mechanism of *HRPT2* gene inactivation, methylation of the promoter, has been reported in 2 of 11 parathyroid carcinomas.(47)

**Table 1:** *HRPT2* Gene Analyses in Parathyroid Carcinomas^*^

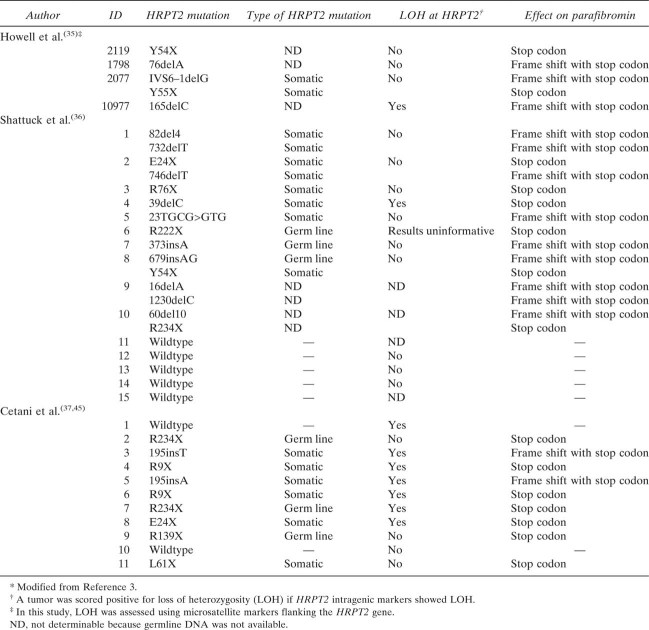

*HRPT2* mutations are found, but only rarely, in sporadic benign parathyroid adenomas.(22,35,37,48) The overall prevalence among the 167 cases of benign disease in which an *HRPT2* mutation has been sought is only 1.8%, or even lower 1/120 (0.8%) if the cases from Carpten et al.,(22) which were selected for cystic features, are excluded. These observations indicate that *HRPT2* mutations have a very limited, if any, role in the pathogenesis of typical sporadic adenomas.

*HRPT2* encodes a protein of 531 amino acids called parafibromin (parathyroid disease and fibro-osseous lesions) that is evolutionarily conserved(22) and similar in homology (54%) to a protein of *Saccharomyces cerevisiae* known as Cdc73. Cdc73 is a component of yeast RNA polymerase II/Paf1 complex, which is involved in the transcription elongation and RNA processing pathways. A human counterpart to the yeast Paf1 complex has been identified.(49–51) Parafibromin is mostly a nuclear protein with a functional bipartite nuclear localization signal (NLS) at residues 125–139 (nucleotides 373–417), which is evolutionary conserved and critical for its nuclear localization.(52–54) Specific *HRPT2* mutations, identified in HPT-JT or sporadic parathyroid carcinoma and predicted to truncate parafibromin upstream of or within the NLS, disrupt nuclear localization.(53) Parafibromin is also localized to the nucleolus. Three nucleolar localization signals at residues 76–92, 192–194, and 393–409 have been identified.(55)

The role of parafibromin as a tumor suppressor protein comes from the observation that parathyroid tumors carrying *HRPT2* mutations are frequently associated with loss of parafibromin expression or function. Transient overexpression of wildtype parafibromin in HEK-293 and NIH3T3 cells, but not its L64P mutant, which is implicated in parathyroid cancer, inhibited cell proliferation by blocking cyclin D1 expression.(56) Thus, it is conceivable that, after biallelic *HRPT2* inactivation, the inhibitory effect of parafibromin on cyclin D1 activity is lost, leading to neoplastic transformation in susceptible tissue such as parathyroid glands.(56) Direct evidence of the anti-proliferative effect of parafibromin was first reported by Zhang et al.(57) in several cell lines by showing that some disease-associated *HRPT2* mutant constructs abolished the ability of wildtype parafibromin to suppress colony formation. Iwata et al.(58) have recently confirmed that the transient overexpression of parafibromin inhibited the growth of HEK293 or NIH3T3 cells. Conversely, in cell lines expressing the large T (LT) antigen, such as 292T and COS-7 cells, transient overexpression of parafibromin increased cell proliferation. Thus, in LT-expressing cells, parafibromin could favor tumorigenesis.

The existence of a connection between parafibromin function and components of the transcription machinery is further supported by the fact that the *Drosophila* ortholog of human parafibromin, hyrax, binds to β-catenin/armadillo and is required for the nuclear transduction of the Wnt/Wingless pathway.(59) Finally, Lin et al.(53) showed that wildtype, but not NLS-mutant, parafibromin promotes apoptosis in transfected cells. Inhibition of endogenous parafibromin expression by RNA interference decreases the basal and cytotoxic-induced apoptosis.

These observations establish the central role of parafibromin in the control of the cell cycle and subsequently in determining cell fate and promoting tumorigenesis. The relationship of parafibromin with the complex network of nuclear components merits further study.

## PATHOLOGY

Parathyroid carcinomas are typically large (>3 cm), irregular, grayish-white, hard tumors often adherent tenaciously to adjacent tissues.(2,4,60) The finding of gross infiltration of contiguous structures strongly suggest the diagnosis of carcinoma. The histological criteria of parathyroid carcinoma are difficult to define and identify. Schantz and Castleman(61) in 1973 established a set of criteria, including thick fibrous bands, mitotic activity, and vascular and capsular invasion. Generally, neoplastic cells (usually chief cells) are arranged in a lobular pattern and separated by dense trabeculae, with mitotic figures. Invasion of the capsule is rather common and, less frequently (10–15%), vascular invasion also occurs.(60) Capsular invasion is characterized by a “tongue-like” protrusion through the collagenous fibers and should be distinguished from pseudoinvasion, because of “trapping” of tumor cells within the capsule, which can be found in adenoma.(2,62) The criteria of vascular invasion have been differently defined according to whether capsular vessels or vessels in the surrounding tissues are involved.(2,62,63) Partial attachment of tumor cells to the wall of the vascular channel or thrombosis should also be present.(2,63)

Because metastatic behavior is rare at presentation,(64) the diagnosis of parathyroid cancer on the basis of the above morphologic criteria may be difficult at the time of the initial operation. Many of the features described above, such as adherence to surrounding tissues, fibrous bands, trabecular growth, and mitosis, are not pathognomonic of malignancy because they can also be found in parathyroid adenomas. The diagnostic value of capsular and vascular invasion is still debated. Some authors regard vascular invasion as virtually diagnostic of malignancy.(2,64) Thus, controversy and diagnostic uncertainties still exist.(2,62,63) The distinction between benign and malignant parathyroid tumors is very hard and rarely made at initial histology. Indeed, in a large series of patients with metastatic parathyroid cancers, as many as 50% were initially classified as benign tumors.(65) A full discussion of the pathology of parathyroid cancer is beyond the scope of this review.

In an attempt to improve diagnostic accuracy, other histological approaches have been studied, but none has yet proven clear diagnostic value.(33,66–72) However, the high rate of *HRPT2* abnormalities in parathyroid carcinomas has paved the way for the development of new diagnostic tools (*HRPT2* mutational status and/or parafibromin immunostaining) of potential utility, particularly in cases with equivocal initial histology (see below). Investigation of patients who have clinically and biochemically severe, but pathologically benign, parathyroid disease, and those with malignant pathology despite mild clinical features will help to elucidate further the utility of these diagnostic tools as markers for parathyroid carcinoma.

## CLINICAL PRESENTATION

The clinical manifestations of parathyroid carcinoma are primarily caused by the effects of markedly elevated serum PTH levels and hypercalcemia rather than by the local infiltration or distant metastases.(1,2)

The typical clinical picture is characterized by signs and symptoms of severe hypercalcemia, with renal involvement (nephrocalcinosis, nephrolithiasis, impaired renal function) in up to 80% of patients, and bone involvement (osteitis fibrosa cystica, subperiosteal resorption, “salt and pepper” skull, diffuse osteopenia) in up to 90%.(1) On physical examination, up to 76% of patients with parathyroid carcinoma have a palpable neck mass.(1) Renal colic is a frequent presenting complaint. Other symptoms include muscle weakness, fatigue, depression, nausea, polydipsia and polyuria, bone pain, and fractures. Recurrent severe pancreatitis, peptic ulcer disease, and anemia can also occur. None of these features is pathognomonic of malignancy. In the majority of cases, the diagnosis of parathyroid carcinoma is made only in retrospect when hypercalcemia recurs because of local spread of tumor or distant metastases. In some patients with parathyroid cancer, a PTH moiety, different from intact PTH(1-84), is produced.(73) The clinical implications of this finding in parathyroid carcinoma await additional studies.

Rarely, parathyroid carcinomas are nonfunctional.(43,74) They can be misdiagnosed as thyroid or thymic carcinoma because of locally advanced disease (palpable neck mass, dysphagia, hoarseness caused by laryngeal nerve palsy). Immunohistochemistry for PTH, thyroglobulin, thyroid transcription factor 1, and calcitonin may help ascertain the correct diagnosis.

## DIFFERENTIAL DIAGNOSIS

### Clinical features

The occurrence of metastases is the only unequivocal criterion of malignancy that is generally accepted, but they usually occur late in the course of the disease.

At initial presentation, despite clinical features suggesting malignancy, it can be a challenge to differentiate between hyperparathyroidism caused by parathyroid carcinoma and that caused by its much more common benign counterpart. Because better outcomes are associated with complete resection of the tumor at the time of initial surgery, it is important to establish the correct diagnosis at the time the patient presents.

Features that might lead to suspect a parathyroid cancer in a patients with PHPT are listed here and in Table 2.

**Table 2:** Clinical Features Useful in the Differential Diagnosis Between Benign and Malignant Primary Hyperparathyroidism^*^

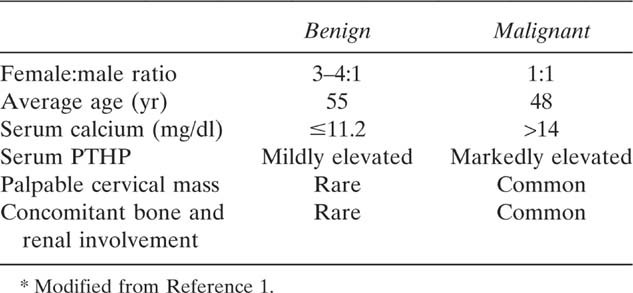

- Male sex: there is no sex preference, whereas the female:male ratio in PHPT favors women by a ratio of 3–4:1
- Relatively young age: the average age of a patient with parathyroid cancer is 50 yr, about 10 yr younger than the usual patient with benign PHPT.
- Markedly elevated serum calcium and PTH: Serum calcium levels are within 1 mg/dl above the upper normal limit in most patients with parathyroid adenomas and >14–15 mg/dl in most patients with parathyroid carcinoma. PTH levels are markedly elevated in patients with parathyroid carcinoma and only slightly elevated in those with adenomas.
- Bone and renal involvement: the combination of both renal and bone manifestations at the time of presentation suggests the possibility of parathyroid carcinoma. In benign PHPT, overt bone disease is unusual, and concomitant skeletal and renal involvement is uncommon.
- Size and appearance of the parathyroid lesion: parathyroid carcinomas are usually >3 cm and may be palpable. The tissue is hard and gray-white and adherent to adjacent structures. Parathyroid adenomas are smaller, dark brown, and firm but not hard.

Alkaline phosphatase activity is also higher in patients with parathyroid carcinoma than in those with adenoma in whom serum levels are generally close to the upper limit of the normal range. α- and β-subunits of human chorionic gonadotropin (hCG) may be elevated in patients with parathyroid cancer but not in those with benign tumors.(75) Urinary hCG levels were found to be elevated in a small group of subjects with parathyroid carcinomas, in contrast to a control group of patients with benign PHPT.(76) In particular, the elevated hCG isotype was the hyperglycosolated form of hCG that is specifically associated with malignancy in trophoblastic and nontrophoblastic diseases. Moreover, elevations of hCG might be predictive of complications such as hip fracture and death.

When benign PHPT presents with markedly elevated serum calcium concentrations and overt target organ involvement, a clinical phenotype that was historically common but is now infrequently seen in most countries, the clinical distinction between benign and malignant disease may be difficult. It is preferable to have a high index of suspicion particularly when concomitant kidney and bone disease are present than to miss the opportunity for surgical cure by failing to consider cancer in the differential diagnosis.

Acute PHPT, sometimes called “parathyroid crisis,” shares many clinical features with parathyroid carcinoma. In view of the marked elevations of serum calcium and PTH that are common in parathyroid crisis, parathyroid cancer should be considered in any differential diagnosis of this condition. Parathyroid cancer should also be considered in any hypercalcemic patient without a history of prior neck surgery who presents with recurrent laryngeal nerve palsy.

### Aids to diagnosis by pathological examination of tissue

Immunohistochemistry is used to improve the accuracy of the diagnosis of parathyroid carcinoma. One approach has involved the use of proliferation markers. Increased labeling of cell cycle–associated antigens (Ki-67, cyclin D1) has been shown in parathyroid carcinoma compared with adenoma,(66,68) but overlap among these tumor types has limited the utility of this approach. Decreased expression of p27, an inhibitor of cyclin-dependent kinase, and abnormal galectin-3 expression have been shown in carcinomas. The association between these abnormalities and high Ki-67 labeling has been suggested to increase the likelihood of malignancy.(70,72)

**FIG. 2:**
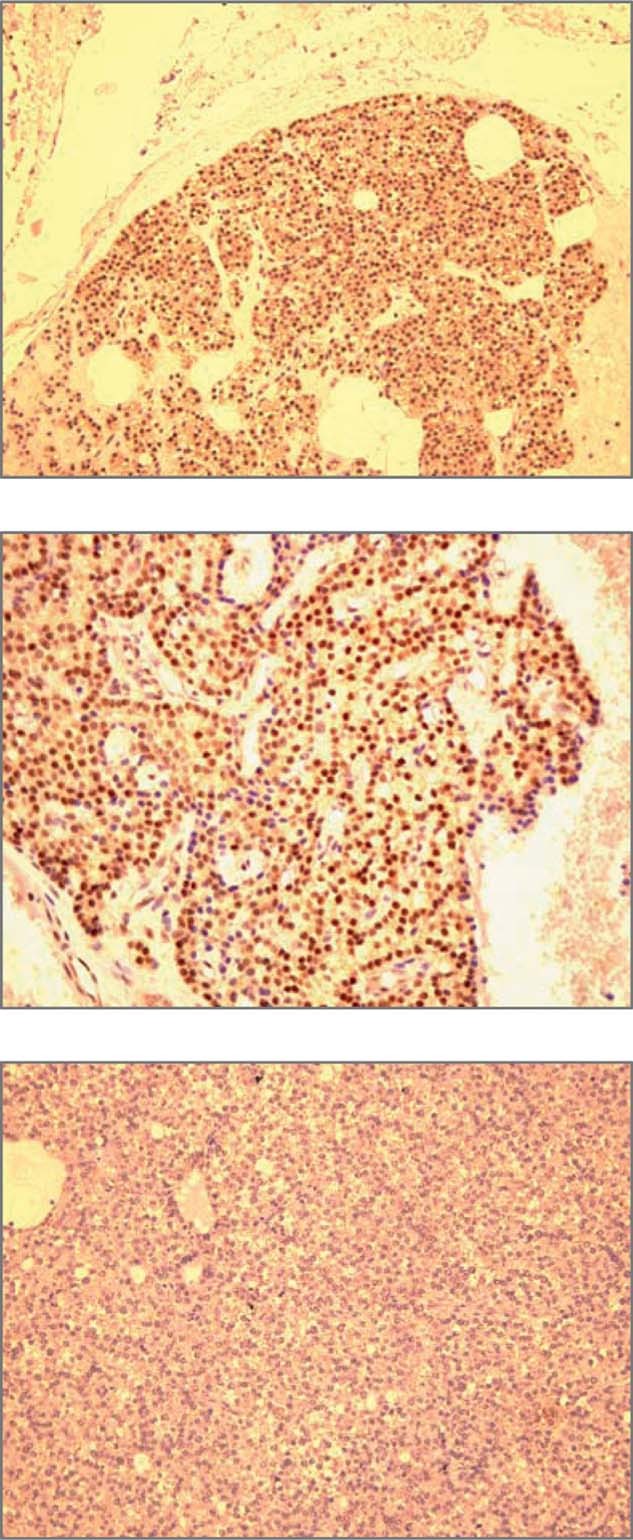
Immunohistochemical analysis of parafibromin expression. (Top) Normal parathyroid. A diffuse nuclear staining is present in most parathyroid cells (×200). (Middle) Parathyroid adenoma. The majority of cells show a positive nuclear staining (×400). (Bottom) Parathyroid carcinoma. Tumor cells show no nuclear staining (×200). (Reproduced from Eur J Endocrinol **156:**547–554 with permission from the European Society of Endocrinology.)

Evaluation of *HRPT2* gene abnormalities seems to be a more promising diagnostic tool.(77) Loss of heterozygosity (LOH) or mutation at the *HRPT2* gene and loss (total or focal) of parafibromin staining have been reported in the large majority of parathyroid carcinomas but very rarely in adenomas(45,78–81) (Fig. 2). To date, limited data are available in equivocal cases, where this technique would have the greatest diagnostic utility (Table 3). It is important to emphasize that the diagnostic potential of these tests hinges on their common presence in parathyroid cancer and their rarity in benign disease. Because benign parathyroid disease is so much more common than parathyroid cancer, a test that has a detectable detection rate in benign parathyroid disease (even if low) would have limited clinical utility. The positive predictive value of the test may be increased, therefore, if the *HRPT2* gene/parafibromin analysis is restricted to cases that are equivocal. The combined findings of negative parafibromin staining and *HRPT2* gene abnormalities increase the likelihood of a malignancy.(45,82) Based on this reasoning, it seems appropriate to evaluate all parathyroid tumors in which the diagnosis is uncertain for abnormalities of both the *HRPT2* gene and its product, parafibromin.

**Table 3:** Summary of Parafibromin Immunohistochemistry in Parathyroid Tumors

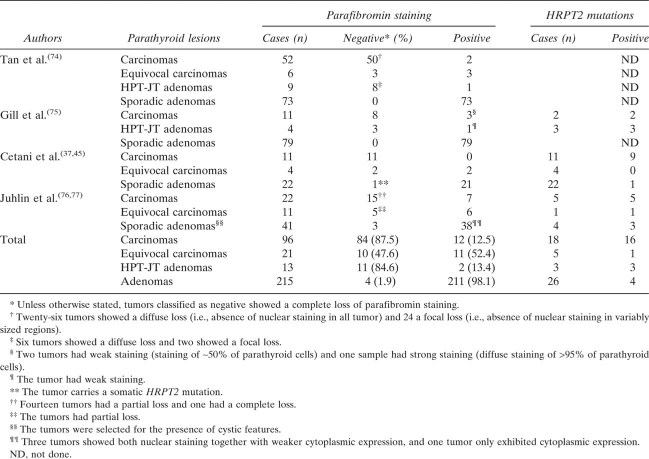

## NATURAL HISTORY AND SURVEILLANCE

Parathyroid carcinoma typically runs an indolent, albeit progressive, course because the tumor has a rather low malignant potential. At initial presentation, very few patients show involvement of regional lymph nodes (<5%) or distant sites (<2%).(1,60) Parathyroid carcinoma recurs locally and spreads to contiguous structures in the neck. Metastases occur late in the course of the disease with spread to cervical nodes (30%) and lung (40%), followed in frequency by liver metastases (10%). Rarely, distant metastases occur in bone, pleura, pericardium, and pancreas.

The identification of *HRPT2* mutations in eight patients with apparently sporadic parathyroid cancers as germline events(36,37,45,46) suggests that a subset of these patients might have HPT-JT syndrome or variant thereof. This observation has implications for the management of recurrent disease in parathyroid cancer. When a patient develops a recurrence of parathyroid cancer, in addition to the likelihood that the original carcinoma has progressed, a new tumor should be carefully sought, because additional, discrete parathyroid tumors can develop in patients with HPT-JT syndrome. Surveillance for renal and jaw lesions is also indicated. Moreover, the relatives of a patient with seemingly sporadic parathyroid carcinoma carrying a germline *HRPT2* mutation are susceptible to the development of parathyroid cancer or other manifestations of HPT-JT syndrome.(43,44) In one such patient, a parathyroid cancer was imaged early by neck ultrasonography in an individual who had not yet become hypercalcemic.(43) Therefore, monitoring of family members with serum calcium determinations and neck ultrasonography is warranted. As suggested by Kelly et al.,(44) surgery should be aimed at identifying and examining all parathyroid glands and en bloc removal of any abnormal tissue. A metal-clip marking the glands left in situ may also be considered, anticipating the need for future surgery. Because of parathyroid cancer in this setting has incomplete penetrance (i.e., not all subjects harboring the *HRPT2* gene abnormality will express the disease), prophylactic total parathyroidectomy is generally not recommended. In those who do undergo complete parathyroidectomy, autotransplantation of parathyroid tissue to the forearm is not recommended to avoid the introduction of potentially malignant tissue at an ectopic site.(44)

## MANAGEMENT

### Surgery

Surgery is the only curative treatment for parathyroid carcinoma and consists of complete resection of the primary lesion at the time of initial operation.(1,5) For this reason, both preoperative suspicion and intraoperative recognition are of great importance. Patients who present with features suggestive of parathyroid carcinoma warrant thorough exploration of all four parathyroid glands, because parathyroid carcinoma has been reported to coexist along with benign adenomas or hyperplasia.(14) The most effective surgical approach is en bloc resection.(83,84) Tracheoesophageal, paratracheal, and upper mediastinal lymph nodes should be excised, but an extensive lateral neck dissection is indicated only when there is spread to the anterior cervical nodes.

When the diagnosis of parathyroid cancer is made after parathyroid surgery on the basis of pathology, as often happens, the management plan becomes more complex. If the macroscopic characteristics of the tumor are typical of a parathyroid carcinoma, and the pathology shows extensive vascular or capsular invasion or if hypercalcemia persists, further exploration of the neck can be considered after appropriate localization studies (see below). The structures surrounding the tumor should be excised as described above. When telling histologic features are absent, the patient is normocalcemic and the diagnosis is only based on equivocal pathology, immediate reoperation is not indicated, because the simple complete resection of the tumor may turn out to be curative. However, such patients should be monitored closely with regular measurement of serum calcium and PTH levels.

Despite a potentially curative resection, parathyroid carcinoma has a recurrence rate of >50%. Most recurrences occur 2–3 yr after the initial operation, but this period is variable, and a prolonged disease-free interval of as long as 20 yr has been reported.(1,85) Imaging studies should be performed in all patients before reoperation. Fine-needle aspiration of a suspicious lesion with measurement of PTH in the eluate should be used with caution, if at all, to avoid seeding the needle track with deposits of malignant cells.(86,87) If noninvasive imaging approaches are negative, arteriography and selective venous sampling for PTH measurement may be useful. The management of recurrent or metastatic parathyroid carcinoma is primarily surgical.(1,5,83–85,88,89) Recurrences in the neck should be treated with wide resections, including the regional lymph nodes and other involved structures. Accessible distant metastases, particularly in the presence of localized metastatic disease, should also be excised, if possible.(1,90) Even a small tumor may produce a sufficient amount of PTH to cause hypercalcemia. Although resection of single metastasis or other foci of malignant tissue is rarely curative, their removal may result in periods of normocalcemia ranging from months to years.(5) Decreasing tumor mass may also render the patient's hypercalcemia more amenable to medical treatment.

### Chemotherapy

Chemotherapy generally is disappointing. Several regimens have been attempted (nitrogen mustard, vincristine, cyclophosphamide, and actinomycin D, and adriamycin alone or in combination with cyclophosphamide and 5-fluorouracil), but none of them has proved to be effective.(91,92) At this time, chemotherapy has no role in the management of patients with parathyroid carcinoma.

### Radiotherapy

With the exception of a single report(91) of an apparent cure (10 yr) in a patient with tumor invasion of trachea, radiation therapy has little, if any, effect in invasive parathyroid cancer.(85) Recent reports have suggested the use of irradiation as adjuvant therapy. The Mayo Clinic reported a disease-free survival at a median follow-up period of 60 mo in four patients who received postoperative adjuvant radiotherapy.(93) The MD Anderson Cancer Center experience also suggests a lower local recurrence rate if adjuvant radiation was given after surgery, independent of the type of operation and the disease stage.(94,95)

### Management of hypercalcemia

When parathyroid carcinoma has became widely metastatic and surgical options are exhausted, clinical management becomes a matter of controlling the hypercalcemia.(96) Saline infusion and loop diuretics are often used, but in the majority of cases, drugs that inhibit bone resorption are needed. Potent intravenous bisphosphonates (pamidronate and zoledronate) may transiently control hypercalcemia, but patients frequently become refractory to them. Plicamicin is effective, but the response is transient, and repeated courses may be associated with toxicity. Octreotide, the long-acting somatostatin analog, has been reported to inhibit PTH secretion in two cases of metastatic parathyroid carcinoma.(97,98)

Anti-PTH immunotherapy showed promise in two recent case reports.(99–101) Dendritic cell immunotherapy may also be applicable to induce a T-cell immune response.(102)

Another approach is to target the parathyroid calcium-sensing receptor (CaSR). Calcimimetics, allosteric modulators of the CaSR, directly reduce parathyroid cell hormone secretion by binding to sites that increase the receptors' affinity for calcium.(103) Thus, sensitivity to extracellular calcium is enhanced. A first-generation calcimimetic, R-568, was used for 2 yr in a patient with metastatic parathyroid cancer with controlled hypercalcemia.(104) R-568 has been replaced by cinacalcet, a more potent second-generation agent with a longer half-life and more predictable hepatic metabolism. In benign PHPT, cinacalcet normalizes serum calcium and partially reduces PTH concentrations for up to 3 yr.(105)

**FIG. 3:**
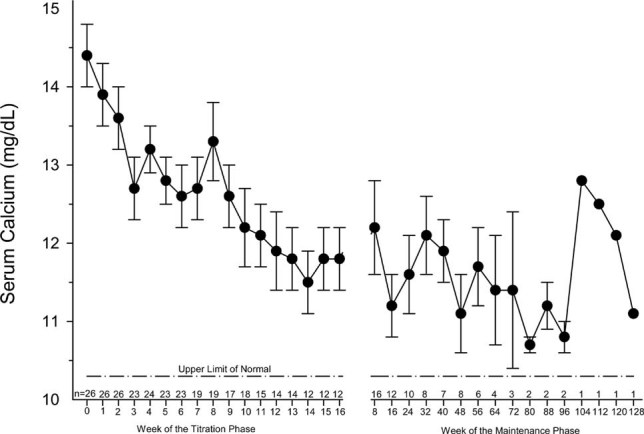
Reduction in serum calcium concentration in parathyroid cancer with cincalcet. Subjects were given cinacalcet in increasing doses, up to 90 mg four times daily, during the titration phase. The average serum calcium fell from 14.5 ± 0.4 to 12.4 ± 0.4 mg/dl (*p* = 0.001). (Adapted from Silverberg SJ, Rubin MR, Faiman C, Peacock M, Shoback DM, Smallridge RC, Schwanauer LE, Olson KA, Klassen P, Bilezikian JP 2007 Cinacalcet hydrochloride reduces the serum calcium concentration in inoperable parathyroid carcinoma. J Clin Endocrinol Metab **92:**3803–3808, *Copyright 2007, The Endocrine Society.*)

Recently, the results of a multicenter study of cinacalcet in 29 patients with inoperable parathyroid carcinoma were published.(106) The primary endpoint of the study was the proportion of patients experiencing a ≥1-mg/dl reduction in serum calcium from baseline at the end of the titration phase. Secondary endpoints included changes from baseline in serum calcium, plasma PTH, bone turnover markers, and health-related quality of life variables. The dose of cinacalcet in this study was titrated from 30 mg twice daily (a dose that might be effective in benign PHPT) up to 90 mg four times daily as required to lower serum calcium levels. Duration of treatment ranged from 1 to 1051 days (mean, 328 ± 306 days). Cinacalcet effectively reduced hypercalcemia in about two thirds of patients with inoperable parathyroid carcinoma (Fig. 3). In the responders (18 of 29 patients), serum calcium levels declined from 15.0 ± 0.5 to 11.2 ± 0.3 mg/dl (*p* < 0.001), with the greatest responses seen in those patients with the highest levels of serum calcium at study entry. It was of interest that the marked reductions in serum calcium were not accompanied by a similar fall in circulating PTH. PTH levels reached a nadir 4 h after drug administration, but the decline was not pronounced, nor was it sustained. Although hypotheses abound, the reason for the discrepancy in calcium and PTH response to cinacalcet remains unclear at this time. Nausea and vomiting were the most common adverse events. Reported in more than one half of all patients receiving cinacalcet at these doses, these symptoms necessitated discontinuation of drug in some cases. Serious adverse events, including fracture and death, were not considered to be drug related. Instead, they were expected consequences of the patients' longstanding, often widely metastatic, underlying disease. There are no data suggesting that cinacalcet alters the course of the parathyroid cancer itself. Therefore, this agent should not be introduced to control hypercalcemia in a patient with a metastatic lesion that is accessible and amenable to surgical extirpation. However, the data do suggest that cinacalcet is useful in reducing calcium levels, and is tolerated in many patients at cumulative doses up to 360 mg/d. Furthermore, unlike other options for treatment of hypercalcemia, this agent can be used in patients with the renal impairment so common in patients with longstanding parathyroid cancer. Cinacalcet therefore represents an important new option for management of intractable hypercalcemia in patients with inoperable disease.

## PROGNOSIS

The prognosis of parathyroid carcinoma is quite variable. No single characteristic correlates with outcome. The best prognosis depends on early recognition and complete excision of the tumor at initial surgery. The mean time to recurrence is usually 3 yr, although intervals of up to 20 yr have been reported.(1,65) Once the tumor recurs, complete cure is unlikely, although prolonged survival is still common with palliative surgery. Five-year survival rates vary from 40% to 86%. The National Cancer Database survey reported a 10-yr survival of ∼49%,(107) and the MD Andersen Cancer Center reported survival rates of 85% and 77% at 5 and 10 yr, respectively.(89) The National Surveillance, Epidemiology, and End Results database recently reported a 10-yr survival of 67.8%.(108)

## SUMMARY

The best opportunity to cure parathyroid carcinoma is to diagnose it before or at the time of parathyroid surgery and for the tumor to be completely removed at the time of the initial operation. Because the diagnosis is often not clear at the time of presentation, recent attempts to distinguish between benign and malignant disease both by genetic and immunohistological analyses are promising. The disease is indolent but progressive. Attempts to remove local recurrences and distant metastases can provide short- and long-term control. Other therapeutic approaches with chemotherapy and radiotherapy are not helpful. Available medical therapy targets the consequence of the disease (hypercalcemia) rather than the disease itself.
